# MicroRNA‐30c suppresses the pro‐fibrogenic effects of cardiac fibroblasts induced by TGF‐β1 and prevents atrial fibrosis by targeting TGFβRII

**DOI:** 10.1111/jcmm.13548

**Published:** 2018-03-13

**Authors:** Juan Xu, Haiqing Wu, Songwen Chen, Baozhen Qi, Genqing Zhou, Lidong Cai, Liqun Zhao, Yong Wei, Shaowen Liu

**Affiliations:** ^1^ Department of Cardiology Shanghai General Hospital Shanghai Jiao Tong University School of Medicine Shanghai China; ^2^ Department of Cardiology Shanghai Institute of Cardiovascular Disease Zhongshan Hospital Fudan University Shanghai China; ^3^ Department of Cardiology Shanghai Songjiang Central Hospital Shanghai China

**Keywords:** atrial fibrosis, miR‐30c, TGFβRII

## Abstract

Atrial fibrosis serves as an important contributor to atrial fibrillation (AF). Recent data have suggested that microRNA‐30c (miR‐30c) is involved in fibrotic remodelling and cancer development, but the specific role of miR‐30c in atrial fibrosis remains unclear. The purpose of this study was to investigate the role of miR‐30c in atrial fibrosis and its underlying mechanisms through in vivo and in vitro experiments. Our results indicate that miR‐30c is significantly down‐regulated in the rat abdominal aortic constriction (AAC) model and in the cellular model of fibrosis induced by transforming growth factor‐β1 (TGF‐β1). Overexpression of miR‐30c in cardiac fibroblasts (CFs) markedly inhibits CF proliferation, differentiation, migration and collagen production, whereas decrease in miR‐30c leads to the opposite results. Moreover, we identified TGFβRII as a target of miR‐30c. Finally, transferring adeno‐associated virus 9 (AAV9)‐miR‐30c into the inferior vena cava of rats attenuated fibrosis in the left atrium following AAC. These data indicate that miR‐30c attenuates atrial fibrosis via inhibition of CF proliferation, differentiation, migration and collagen production by targeting TGFβRII, suggesting that miR‐30c might be a novel potential therapeutic target for preventing atrial fibrosis.

## 1 INTRODUCTION

Atrial fibrillation (AF) is the most common sustained cardiac arrhythmia, and it results in significant morbidity and mortality.^1^ Atrial fibrosis plays a key role in the development and maintenance of AF.^2^, ^3^

MicroRNAs (miRNAs) are small non‐coding RNAs that play important roles in regulating gene expression, resulting in either translation repression or mRNA degradation through actions on the 3′untranslated region (3′UTR) of target genes.^4^ MiRNAs participate in the pathogenesis of different pathological events, including cardiac fibrosis.^5^ Several members of the miR‐30 family are highly expressed in the brain, with the exception of miR‐30c, which is abundant in cardiac fibroblasts (CFs) and is involved in ventricular fibrosis.^6^, ^7^ Several studies have demonstrated that miR‐30c inhibits ventricular collagen synthesis in pathological left ventricular hypertrophy.^7^, ^8^ Roy et al^9^ indicated that miR‐30c modifies the transforming growth factor‐β (TGF‐β)‐dependent regulation of extracellular matrix (ECM)‐related genes in HSCs during CCl_4_‐induced liver fibrosis. Wang et al^10^ suggested that miR‐30c suppresses renal fibrosis and improves renal function. Taken together, these data suggest that miR‐30c is an anti‐fibrotic miRNA mediating organ fibrosis. However, it remains unclear whether miR‐30c is involved in atrial fibrosis and through what underlying mechanisms.

Fibrosis is a complex process resulting from activation of select signalling pathways, such as TGF‐β1 signalling.^11^ TGF‐β1 is essential for the development of atrial fibrosis.^12^, ^13^, ^14^ The levels of TGF‐β1 are increased in the plasma and atria of AF patients.^15^, ^16^ Moreover, animal models have revealed that transgenic overexpression of TGF‐β1 results in atrial‐specific fibrosis and increases AF susceptibility.^17^, ^18^, ^19^ Thus, we used TGF‐β1‐induced CFs to establish an in vitro model of atrial fibrosis. TGF‐β1 mediates its effects via interactions among transmembrane receptors, including type I (TGFβRI), type II (TGFβRII) and type III (TGFβRIII). Upon binding to TGF‐β1, TGFβRII recruits and phosphorylates TGFβRI, which initiates signal transduction via Smad proteins.^20^ TGFβRII can transduce the TGF‐β1 signalling from cell membrane to the cytoplasm and then regulate a series of physiological or pathological processes including cell proliferation, differentiation 21, 22 and collagen production.^23^ We identified TGFβRII as a direct target of miR‐30c using a microRNA target prediction tool (RNAhybrid). Recent studies show that TGFβRII is a target gene of several anti‐fibrotic miRNAs. Liang et al^24^ suggested that miR‐153, an anti‐fibrotic miRNA, inhibits the migration of pulmonary fibroblasts and reduces pulmonary fibrosis by targeting TGFβRII. As shown in the study by Zou et al^25^, miR‐19a‐3p/19b‐3p inhibits autophagy‐mediated fibrogenesis by targeting TGFβRII. Another study suggested that miR‐145 prevents TGFβ‐dependent ECM accumulation and fibrosis in smooth muscle cells by targeting TGFβRII.^26^ Additionally, a recent study showed that constitutive expression of dominant‐negative TGFβRII in the posterior left atrium in a canine heart failure model could sufficiently attenuate fibrosis‐induced changes in atrial conduction and restitution to decrease AF.^27^

Collectively, these data suggested that miR‐30c and TGFβRII were critical for TGF‐β1‐mediated fibrosis. Therefore, we hypothesize that miR‐30c represses TGFβRII expression, subsequently decreasing CF proliferation, differentiation, migration and collagen synthesis. Our present work aimed to elucidate the effects of miR‐30c on atrial fibrosis and TGF‐β1‐induced fibrosis performed with in vivo and in vitro experimental approaches, and this may provide a potential therapeutic target to prevent atrial fibrosis.

## 2 MATERIALS AND METHODS

### 2.1 Rat model of atrial fibrosis and Adenovirus Injection

All animal protocols in this study were approved by the Animal Care and Use Committee, Research Institute of Medicine, Shanghai Jiao Tong University, in accordance with the Guide for the Care and Use of Laboratory Animals published by the National Institutes of Health (Publication No.85–23, revised 1996). All efforts were paid to minimize animal suffering. An animal model of atrial fibrosis was induced by pressure overload via abdominal aortic constriction (AAC).^28^, ^29^ Fifteen Sprague‐Dawley rats weighing 200‐250 grams were randomly divided into sham (n = 7) and AAC (n = 8) groups. The rats were anaesthetized intraperitoneally with sodium pentobarbital (40 mg/kg). The AAC procedures were as follows: After opening the abdomen, we separated the abdominal aorta from the right renal artery branch above 5 mm, and a 22‐gauge needle was placed next to the abdominal aorta. A suture was securely tied around the needle and the aorta. After ligation, the needle was quickly removed, and the abdominal cavity was closed. The rats in the Sham group underwent an open‐abdomen procedure without AAC. All surgical procedures were performed under sterile conditions. One of the Sham died from deep anesthesia and one of the AAC died from ruptured abdominal aorta. After constriction with 8 weeks, all rats are killed and the atriums of heart were removed for detecting the expressions of miR‐30c and TGFβRII.

Adeno‐associated virus represents an efficient and safe vector for in vivo gene transfer, and serotype 9 is a cardiotropic serotype.^30^, ^31^, ^32^ Thence, AAV9 expressing miR‐30c (AAV9‐miR‐30c) and a negative control (AAV9‐Control) were used to determine whether miR‐30c overexpression is sufficient to reduce atrial fibrosis in AAC models. AAV9‐miR‐30c or AAV9‐Control (Hanheng Biotechnology, China) was randomly injected into inferior vena cava (IVC) with insulin needles, as soon as AAC at 1.8 × 10^11^v.g./200 μl per rat.^31^ The rats were randomly divided into 4 groups: Sham (n = 6), AAC (n = 8), AAC+ miR‐30c (n=8) and AAC+ Control (n=8). After treatment for 8 weeks, excessive anaesthesia, venous thrombosis, resulted in 4 deaths, leaving the number of rats in the 4 groups was Sham (n = 6), AAC (n = 6), AAC + miR‐30c (n = 7) and AAC + Control (n = 7). The left atrium was removed and divided into 2 parts. One part was rapidly frozen in liquid nitrogen for subsequent RNA isolation, protein isolation, and the remaining part was kept in 4% (w/v) paraformaldehyde (PFA) for histological analysis.

### 2.2 Masson's trichrome staining

Atrial tissue samples were fixed with 4% (w/v) PFA for 48 hours, subjected to alcohol dehydration, embedded in paraffin and sliced into 4 μm thick sections, which underwent Masson's trichrome staining to highlight fibres. The percentage of fibrosis was measured as fibrosis areas/total given field areas × 100%.

### 2.3 Cell culture, stimulation and transfection

293T cells were purchased from the American Type Culture Collection (ATCC, Manassas, USA) and were cultured in Dulbecco's modified Eagle medium (DMEM, HyClone, USA) with 10% foetal bovine serum (FBS, Gibco, USA). Primary cultures of neonatal CFs were isolated from the atria of 1‐ to 3‐day‐old Sprague‐Dawley rats as previously described.^33^ Briefly, the tissues were diced into small pieces and carefully washed in ice‐cold phosphate‐buffered saline (PBS, HyClone, USA) to remove plasma contaminants. The pieces were digested with a 0.25% trypsin solution and 0.1% collagenase II solution, until the tissue was no longer visible. The isolated CFs were maintained in DMEM supplemented with 10% FBS and 1% penicillin/streptomycin and incubated in 95% air/5% CO_2_ at 37°C by removing unattached cells (including cardiomyocytes and endothelial cells) after 1.5 hours. The CFs were defined as passage 0 (P0). The cells were digested with 0.25% trypsin‐EDTA for passaging. CFs at passage 2 or 3 (P2‐P3) were used in the following studies. The cells were transfected for 72 hours with miR‐30c mimic, miR‐30c inhibitor and negative controls, according to the Lipofectamine^®^ 3000 (Invitrogen, USA) instructions. The miR‐30c mimic, mimic negative control (miR01201‐1‐5), inhibitor and inhibitor negative control (miR02201‐1‐5) were purchased from RiboBio (Guangzhou, China). The recombinant human TGF‐β1 (5 ng/mL, Peprotech, USA) was used to establish the cellular model of fibrosis. After CF transfection and stimulation for 24 hours, the Opti‐MEM was replaced with DMEM containing TGF‐β1 for another 48 hours. After stimulation, the cells were harvested and real‐time PCR and western blotting assays were performed.

### 2.4 RNA isolation and quantitative Real‐Time PCR

Total RNA was extracted from cultured CFs and left atriums using Trizol reagent (Invitrogen, CA), according to the manufacturer's instructions. Total RNA (500 ng for mRNA or 1000 ng for miRNA) from each sample was subjected to reverse transcription according to the instructions of the cDNA Synthesis Kit (TaKaRa, China) and miRNA First Strand cDNA Synthesis Kit (Sangon, China). The expression levels of mRNA and miR‐30c were analysed according to the instructions of the mRNA SYBR qPCR kit (TaKaRa, China) and miRNAs qPCR Kit (Sangon, China) using the ABI‐7300 Real‐Time PCR Detection System (Applied Biosystems, USA). The mRNA primer sequences used in the study are listed in Table 1. The bulge‐loop^™^ miRNA Primer Sets (one RT primer and a pair of qPCR primers) specific for miR‐30c and U6 were purchased from RiboBio (Guangzhou, China). The levels of mRNA and miR‐30c were normalized to β‐Actin and U6, respectively. Briefly, the cycle threshold (CT) values of each targeting gene were subtracted from the CT values of the housekeeping gene, referring as ▵ct. Target gene ▵▵ct was determined as ▵ct of target gene minus ▵ct of control. The relative expression levels of all genes were calculated basing on the 2^−ΔΔct^. All samples were assayed in triplicate.

**Table 1:** Nucleotide sequences of the qRT‐PCR primers

Gene	Forward (5′–3′)	Reverse (5′–3′)
TGFβRII	GACGGCTCCCTGAACACTAC	AGCAATCCTGCTGACCTCTG
Col I	CAATGGCACGGCTGTGTGCG	CACTCGCCCTCCCGTCTTTGG
Col3α1	TGAATGGTGGTTTTCAGTTCAG	GATCCCATCAGCTTCAGAGACT
α‐SMA	GCTCCAGCTATGTGTGAAGAGG	CAACCATCACTCCCTGGTGTC
Vimentin	CAGTCACTCACCTGCGAAGT	GAGTGGGTGTCAACCAGAGG
β‐Actin	ACCCACACTGTGCCCATCTA	GCCACAGGATTCCATACCCA

### 2.5 Cell proliferation

Cell growth was analysed using cell counting kit‐8 (CCK‐8) solution (Dojindo, Japan) according to the manufacturer's instructions. Briefly, the cells were plated at a density of 5 × 10^3^ cells per well in 96‐well plates and transfected as described above. Then, the medium in each well was substituted with 10 μl CCK‐8 solution for 2 hours. The absorbance at 450 nm was measured using a multimode reader.

### 2.6 Cell migration assays

Cellular migration assays were performed in 24‐transwell permeable supports with an 8.0 μm pore size (Corning, USA). After treatment, 5 × 10^4^ cells were seeded in the upper chamber in serum‐free DMEM. The lower chambers were filled with serum‐free DMEM containing TGF‐β1 (5 ng/mL) as a chemoattractant. The cells were allowed to migrate for 24 hours at 37°C in 5% CO_2_ atmosphere. After incubation, the non‐migratory cells on the top of the membrane were removed with a cotton swab. Membranes containing cells were fixed with methanol and stained with crystal violet (Beyotime, China) for 30‐60 minutes. The number of migrated cells was counted from 5 randomly selected fields at 200 × magnification using a microscope.

### 2.7 Western blot analysis

Briefly, total protein was isolated from ground frozen atrium tissues and CFs using RIPA lysis buffer (Beyotime, China). The samples were clarified by centrifugation at 13 800 g for 15 minutes at 4°C. Protein concentrations were determined using the BCA Protein Assay Kit (Beyotime, China). A total of 20 μg of protein was separated on 10% SDS‐PAGE at 80‐100 V for 2 hours and transferred onto PVDF membranes (Millipore, USA) at 300 mA for 1.5 hours. The membranes were blocked with 5% non‐fat milk in Tris‐buffered saline with tween 20 (TBST) for 1 hour at room temperature. Each membrane was incubated with primary antibodies at 4°C overnight and then secondary antibodies at room temperature for 1 hour the next day. The following antibodies were used: rabbit anti‐TGFβRII (1:500, Santa Cruz), rabbit anti‐α‐SMA (1:1000, Abcam), rabbit anti‐Col I (1:1000, Abcam), rabbit anti‐vimentin (1:1000, Abcam), mouse anti‐Col3α1 (1:250, Santa Cruz) and mouse anti‐GAPDH (1:2000, Abcam) antibodies. All proteins were visualized using the ECL reagent Kit (Millipore Corp, USA). The values of the band intensities were quantified using Image J software.

### 2.8 Dual luciferase activity assay

To investigate the target gene of miR‐30c, bioinformatic prediction algorithms were used to analyse and identify potential targets. In briefly, the 3′UTR of TGFβRII gene was chemically synthesized and inserted at the XbaI site, immediately downstream of the luciferase gene in the pGL3‐promoter vector (Promega, USA) to generate the TGFβRII wild‐type (WT)‐luciferase vector. The TGFβRII mutant (mu)‐luciferase vector was generated using the MutaBest kit (Takara, China). Subsequently, 293T cells were seeded at a density of 2 × 10^4^ cells per well in 24‐well plates. The 293T cells were transfected with 1 μg of the pGL3 luciferase expression construct containing the 3′UTR of TGFβRII, 50 ng of the pRL‐SV40 renilla luciferase vector (Promega, USA), and cotransfection with 50 nmol/L miR‐30c mimic or negative control. After transfection for 48 hours, the luciferase activity was measured using a Dual Luciferase Report Assay Kit (Promega, USA) and normalized to renilla luciferase activity. The following primer sets were used for this study:

3′UTR TGFβRII–WT:

Forward GCTCTTACGCGTGCTAGCCTTTTTCTGGGCAGGCTG

Reverse TGCAGATCGCAGATCTCGAGCTAGGGAAGAAGGGAGC

3′UTR TGFβRII–mu:

Forward AGGAATGGTGCTGAGGTGTGGTCTTTGGTTAGAGGAC

Reverse CACACCTCAGCACCATTCCTTTCCTGACTGATGCTTC

### 2.9 Statistical analysis

Statistical analysis was performed using Graphpad Prism 5 software and SPSS 19.0. The data from all groups were expressed as the mean ± SEM. Statistical comparisons between 2 groups were performed by a *t*‐test. The difference among multiple groups was evaluated using a 1‐way ANOVA followed by Bonferroni post‐tests. A *P* value less than .05 was considered statistically significant.

## 3 RESULTS

### 3.1 miR‐30c is down‐regulated in the rat abdominal aortic constriction model

**Figure 1:**
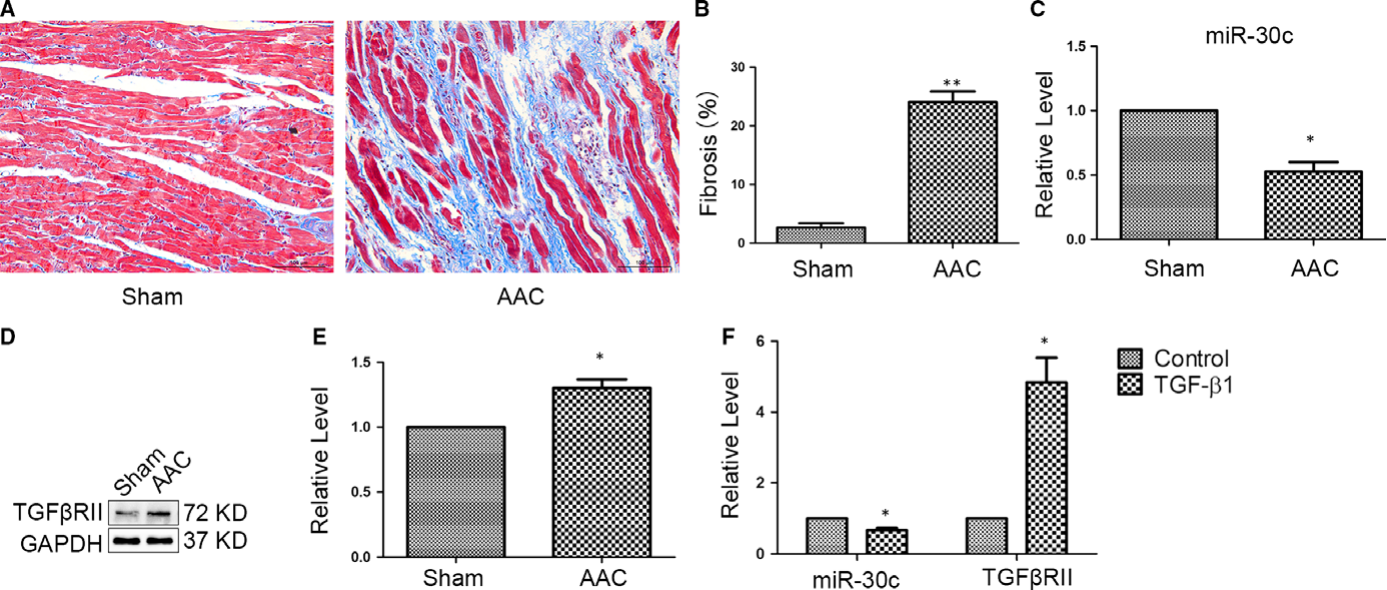
MiR‐30c was decreased in atrial fibrosis. (A, B) Masson's trichrome staining and quantitative analysis in left atrium of Sham (n = 6) and AAC (n = 7) groups. The blue staining indicates interstitial fibrosis. Interstitial fibrosis was increased in AAC rats. **P < .01 compared with the Sham group (Scale bar, 100 μm). (C) The level of miR‐30c was down‐regulated in the left atrium of AAC rat model by qRT‐PCR. *P < .05 compared with the Sham group. (D, E) Representative western blot and quantification analysis revealed that the protein levels of TGFβRII were up‐regulated in left atrium of the AAC rat models. *P < .05 compared with the Sham group. (F) The expression levels of miR‐30c and TGFβRII in CFs with TGF‐β1 (5 ng/mL) were down‐regulated and up‐regulated by qRT‐PCR, respectively. *P < .05 compared with Control group. All experiments were repeated 3 times and data were presented as the mean ± SEM (n = 3, each)

To explore the potential role of miR‐30c in atrial fibrosis, we first established a model of atrial fibrosis by AAC. Based on Masson's trichrome staining, we identified higher collagen production in the AAC group than in the Sham group (Figure 1A,B). In addition, we confirmed that miR‐30c was down‐regulated in atrial samples from AAC model rats via quantitative Real‐Time PCR (qRT‐PCR) (Figure 1C). Furthermore, the level of miR‐30c was also decreased in CFs stimulated with TGF‐β1 (Figure 1F). Taken together, these data supported a potential role for miR‐30c for atrial fibrosis. The TGFβRII expressions were increased both in AAC models and cultured neonatal rat CFs stimulated with TGF‐β1 (Figure 1D–F).

### 3.2 miR‐30c reduces TGF‐β1‐induced CF proliferation, differentiation and migration

**Figure 2:**
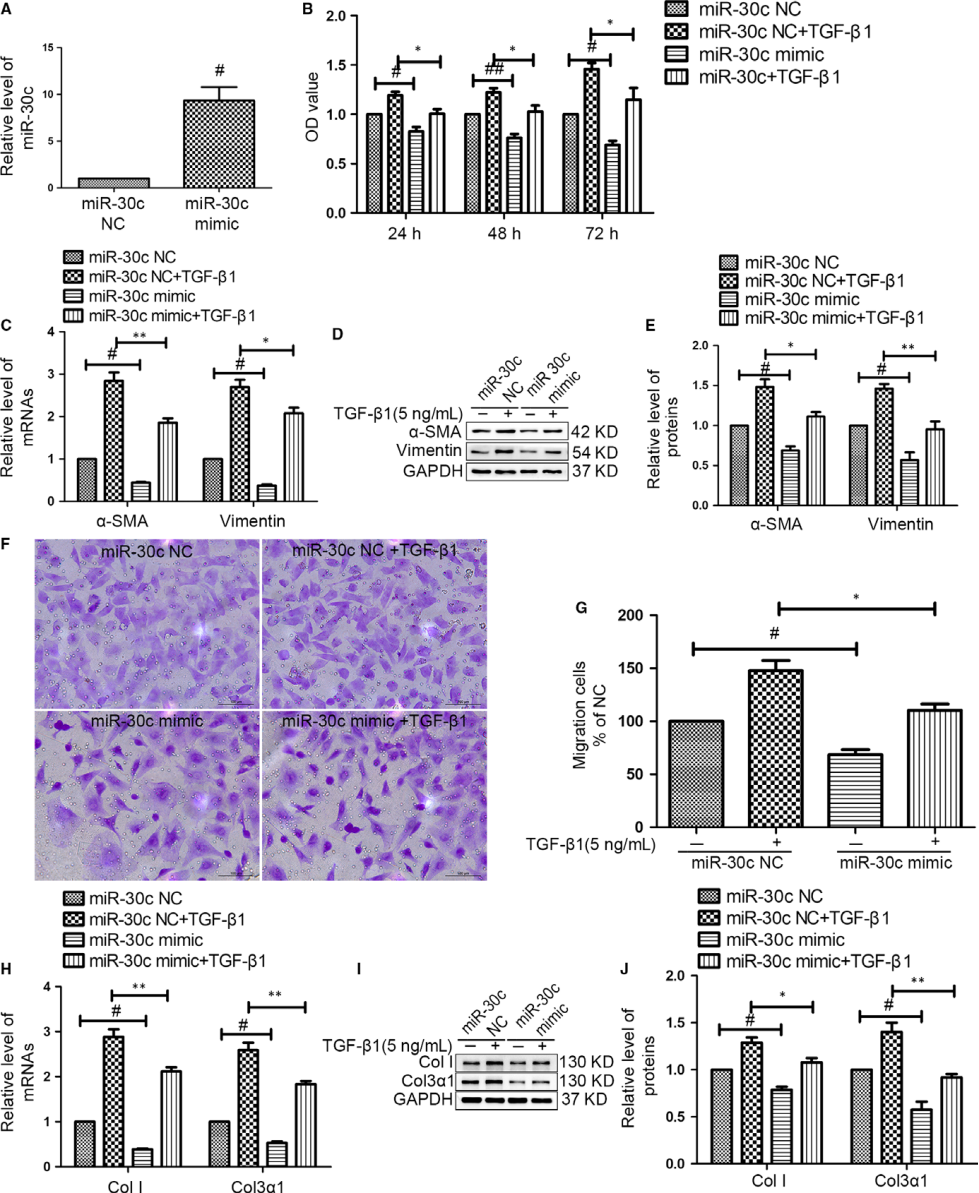
MiR‐30c exhibited an anti‐fibrotic effect on CFs. (A) CFs were transfected with a miR‐30c mimic or miR‐30c negative control (NC) for 72 hours. NC was used as a mimic negative control. The level of miR‐30c was increased in CFs transfected with miR‐30c mimic by qRT‐PCR. ^#^ *P* < .05 compared with NC group. (B) After transfecting with a miR‐30c mimic or NC, the CFs were stimulated with TGF‐β1 (5 ng/mL) for 24, 48 or 72 hours. Overexpression of miR‐30c inhibited CF proliferation performed with a CCK8‐Kit assay. ^#^ *P* < .05, ^##^ *P* < .01 compared with NC group; *P < .05 compared with NC + TGF‐β1 group. (C–E) qRT‐PCR, western blot and quantification analysis of levels of α‐SMA and vimentin in CFs transfected with the miR‐30c mimic or negative control in the presence or absence of TGF‐β1 (5 ng/mL). ^#^ *P* < .05 compared with NC group; *P < .05, **P < .01 compared with NC + TGF‐β1 group. (F) Representative photomicrographs of the migrating cells after transfection and staining by crystal violet (Scale bar, 100 μm). (G) The effect of the miR‐30c mimic on the migration of the cells was quantified. ^#^ *P* < .05 compared with NC group; *P < .05 compared with NC + TGF‐β1 group. (H–J) The mRNA and protein levels of Col I and Col3α1 in CFs transfected with the miR‐30c mimic were decreased in the presence or absence of TGF‐β1 (5 ng/mL) via qRT‐PCR, representative western blot and quantitative analysis for 72 hours. ^#^ *P* < .05 compared with NC group; *P < .05 and **P < .01 compared with NC + TGF‐β1 group. All experiments were repeated 3 times, and all data were presented as the mean ± SEM (n = 3, each)

Next, we investigated the effect of miR‐30c on CF proliferation, differentiation migration and ECM‐related proteins in vitro. Based on the qRT‐PCR analysis, we confirmed that miR‐30c was successfully up‐regulated after transfected with miR‐30c mimic for 72 hours (Figure 2A). First, we observed that miR‐30c mimic significantly inhibited CF proliferation at 24, 48 and 72 hours with or without TGF‐β1 via CCK‐8 assay compared with that in negative control group (Figure 2B). In addition, the overexpressing miR‐30c attenuated the differentiation of CFs into myofibroblasts as indicated by decreased the levels of vimentin and α‐smooth muscle actin (α‐SMA) (Figure 2C–E), which is a reliable and classic marker for the myofibroblast.^33^, ^34^, ^35^ Furthermore, our result demonstrated that a miR‐30c mimic inhibited CF stimulated with or without TGF‐β1 migration via transwell migration assay (Figure 2F,G). Similarly, overexpression of miR‐30c inhibited the increased mRNA and protein levels of collagen I (Col I) and collagen3α1 (Col3α1) with or without TGF‐β1 (Figure 2H–J).

**Figure 3:**
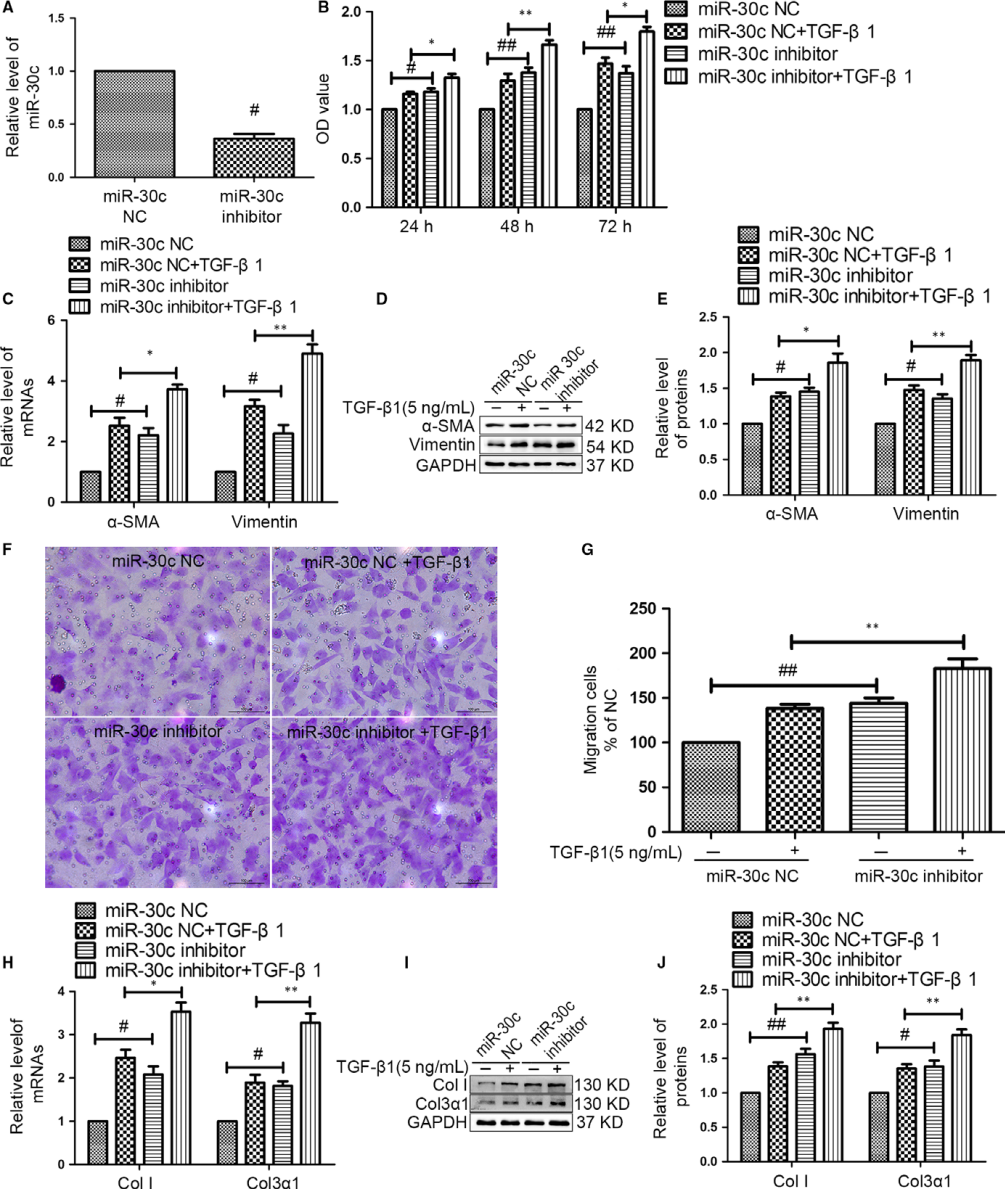
The miR‐30c inhibitor reversed the anti‐fibrotic effects on CFs. (A) CFs were transfected with a miR‐30c inhibitor or miR‐30c inhibitor negative control (NC) for 72 hours. The level of miR‐30c was decreased in CFs transfected with miR‐30c inhibitor by qRT‐PCR. NC was used as an inhibitor negative control. ^#^ *P* < .05 compared with NC group. (B) After transfecting with a miR‐30c inhibitor or NC, the CFs were stimulated with TGF‐β1 (5 ng/mL) for 24, 48 or 72 hours. A miR‐30c inhibitor promoted CF proliferation evaluating by a CCK8‐Kit assay. ^#^ *P* < .05, ^##^ *P* < .01, compared with NC group; *P < .05, **P < .01, compared with NC + TGF‐β1 group. (C–E) The levels of α‐SMA and vimentin were increased in CFs transfected with the miR‐30c inhibitor in the presence or absence of TGF‐β1 (5 ng/mL) via qRT‐PCR, western blot and quantification analysis. ^#^ *P* < .05 compared with NC group; *P < .05, **P < .01, compared with NC + TGF‐β1 group. (F) Representative photomicrographs of the migrating cell after transfection and staining by crystal violet (Scale bar, 100 μm). (G) The effect of the miR‐30c inhibitor on the migration of the cells was quantified. ^##^ *P* < .01, compared with NC group; **P < .01 compared with NC + TGF‐β1 group. (H–J) qRT‐PCR, representative western blot and quantitative analysis of the mRNA and protein levels of Col I and Col3α1 in CFs transfected with the miR‐30c inhibitor or NC in the presence or absence of TGF‐β1 (5 ng/mL) for 72 hours. A miR‐30c inhibitor increased the levels of Col I and Col3α1. *P < .05, **P < .01, compared with NC + TGF‐β1 group; ^#^ *P* < .05, ^##^ *P* < .05, compared with NC group. All experiments were repeated 3 times, and all data were presented as the mean ± SEM (n = 3, each)

Then, we inhibited miR‐30c expression by transfecting the cells with miR‐30c inhibitors. Based on the qRT‐PCR data, miR‐30c expression was successfully inhibited in CFs (Figure 3A). MiR‐30c inhibitor increased CF proliferation with or without TGF‐β1 compared with that in negative control group at 24, 48 and 72 hours via CCK‐8 assay (Figure 3B). In addition, the a‐SMA and vimentin levels were significantly increased in the miR‐30c inhibitor + TGF‐β1 group at 72 hours (Figure 3C–E). MiR‐30c inhibitor promotes CF migration (Figure 3F, G). Contrary to the effects of miR‐30c overexpression, inhibition of miR‐30c increased the expression levels of Col I and Col3α1 (Figure 3H–J). Collectively, these data indicated that overexpression of miR‐30c attenuates CF proliferation, differentiation and migration and collagen synthesis.

### 3.3 miR‐30c targets TGFβRII

**Figure 4:**
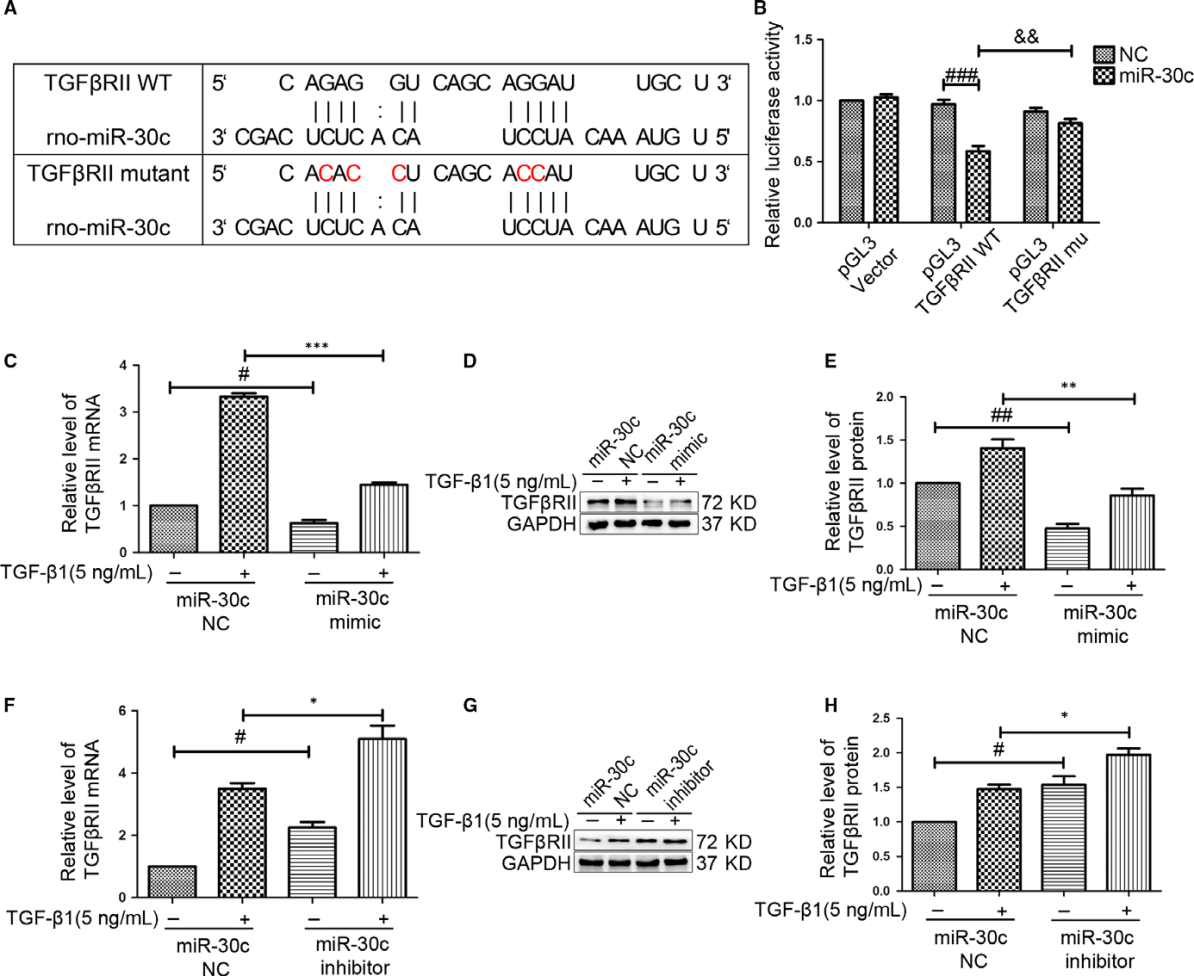
MiR‐30c targets TGFβRII. (A) Diagram of miR‐30c binding site in TGFβRII 3′UTR. TGFβRII 3′UTR mutant was constructed to evaluate miR‐30c binding. (B) After 293T cell cotransfection, luciferase activity was measured using a Dual Luciferase Report Assay Kit. The luciferase activity was significantly decreased in TGFβRII‐WT + miR‐30c group compared with that in TGFβRII‐WT + miR‐30c NC and TGFβRII‐mu + miR‐30c. ^###^ *P* < .001, compared with pGL3‐TGFβRII‐WT + NC group. ^&&^ *P* < .01, compared with pGL3‐TGFβRII‐mu + miR‐30c group. (C–E) qRT‐PCR and western blot analyses of CFs transfected with the miR‐30c mimic or negative control for 72 hours in the absence or presence of TGF‐β1. The miR‐30c mimic decreased the mRNA and protein levels of TGFβRII. ^#^ *P* < .05, ^##^ *P* < .05 compared with NC group; **P < .01, ***P < .01, compared with NC + TGF‐β1 group. (F–H) The level of TGFβRII was increased after transfection with a miR‐30c inhibitor as measured by qRT‐PCR and western blot. ^#^ *P* < .05 compared with NC group; *P < .05 compared with NC+TGF‐β1 group. All experiments were repeated 3 times, and all data were presented as the mean ± SEM (n = 3, each)

According to the above results, we verified that miR‐30c was involved in CF proliferation, differentiation and migration. Further, we wondered about the potential relationship between miR‐30c and TGFβRII. Figure 4A shows the proposed microRNA binding site in 3′UTR of TGFβRII gene as well as mutant 3′UTR that was generated in this study. The luciferase activity was significantly decreased in pGL3‐TGFβRII‐WT + miR‐30c group compared with that in pGL3‐TGFβRII‐mu + miR‐30c group (Figure 4B). For further verification, we confirmed that up‐ and down‐regulation of miR‐30c in CFs could decrease and increase the mRNA and protein levels of TGFβRII after transfection with a miR‐30c mimic or a miR‐30c inhibitor in the absence or presence of TGF‐β1 (Figure 4C–H). These outcomes verified that miR‐30c could target TGFβRII.

### 3.4 miR‐30c prevents AAC‐induced atrial fibrosis

**Figure 5:**
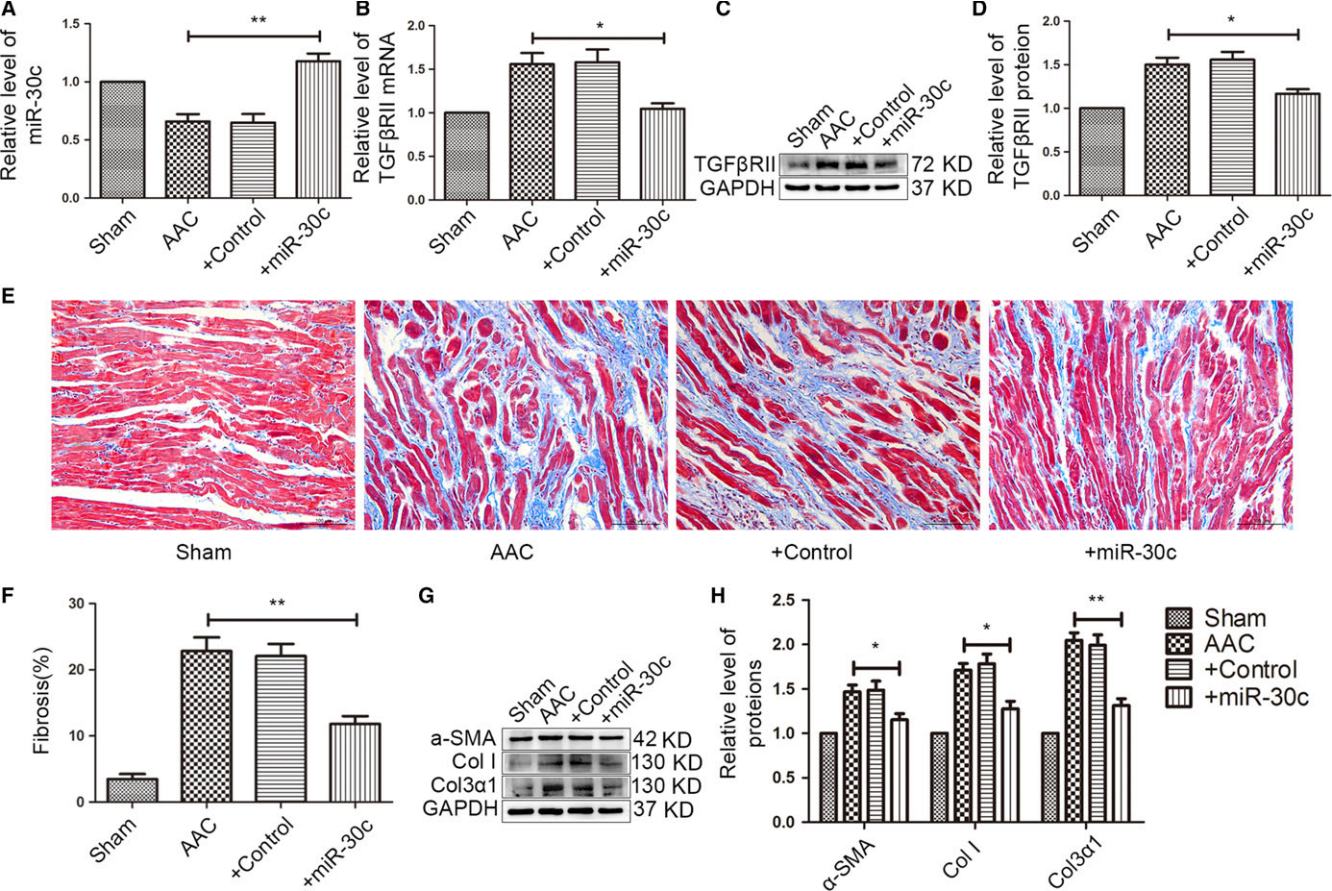
In vivo overexpression of miR‐30c via AAV9 attenuates atrial fibrosis. (A) After administering AAV9‐miR‐30c into the inferior vena cava for 8 weeks, the level of miR‐30c in the left atrium was increased by qRT‐PCR. **P < .01, compared with AAC group. (B–D) The levels of TGFβRII were determined by qRT‐PCR and western blot. The mRNA and protein levels of TGFβRII were decreased in the AAC+miR‐30c group. *P < .05, compared with AAC group. (E, F) Masson's trichrome staining and quantitative analysis in left atrium demonstrated that overexpression miR‐30c prevented atrial fibrosis (Scale bar, 100 μm). **P < .01, compared with AAC group. (G, H) The protein levels of Col I, Col3α1 and α‐SMA were measured by western blot. In vivo, overexpression miR‐30c inhibited the expression of Col I, Col3α1 and α‐SMA. *P < .05, **P < .01, compared with AAC group. (Sham, n = 6; AAC, n = 6; AAC + miR‐30c, n = 7; AAC + Control, n = 7)

To further determine whether miR‐30c indeed plays a vital role in alleviating atrial fibrosis induced by AAC, we next used a heart‐specific AAV9 via IVC injection to achieve overexpression of miR‐30c in left atrium. We confirmed that miR‐30c expression was efficiently increased in the left atrium of rats in the AAC + miR‐30c group via qRT‐PCR (Figure 5A). In addition, the mRNA and protein expressions of TGFβRII were decreased in the AAC + miR‐30c group (Figure 5B–D). Up‐regulation of the miR‐30c could attenuate the growth of fibres induced by AAC via Masson's trichrome staining (Figure 5E,F). Moreover, administration of miR‐30c substantially inhibited the expression of Col I, Col3α1 and α‐SMA, compared with those in AAC group (Figure 5G,H). These data provide strong evidence that up‐regulation of miR‐30c has a cardio‐protective effect against fibrosis and that miR‐30c inhibits TGFβRII expression alleviating left atrial fibrosis.

## 4 DISCUSSION

In this study, we presented several novel findings. First, the miR‐30c expression was decreased with the progression of atrial fibrosis. In addition, overexpression of miR‐30c suppressed TGF‐β1‐induced CF proliferation, differentiation, migration, synthesis of ECM and TGFβRII expression. In contrast, a miR‐30c inhibitor reversed this anti‐fibrotic effect. Moreover, a dual luciferase reporter assay indicated that TGFβRII is a target gene of miR‐30c. Our results further confirmed that exogenous miR‐30c attenuated atrial fibrosis under pathological conditions.

Atrial fibrosis is associated with cellular alterations such as proliferation, migration and fibroblast‐to‐myofibroblast transition, leading to an increase in the volume of dysfunctional ECM. The link between ECM remodelling and AF maintenance is now universally recognized. As a result, atrial non‐homogeneity in conduction contributes to the maintenance and progression of AF.^36^, ^37^, ^38^ Our previous study clearly indicated that permanent ventricular pressure overload by AAC induces atrial remodelling, including hypertrophy, dilatation and fibrosis, and susceptibility to AF.^39^ This is consistent with other observations that AAC induced atrial fibrosis and increased vulnerability to AF.^28^, ^40^ Atrial interstitial fibrosis provides a better understanding of the initiation, maintenance and progression of AF.

Accumulating evidence has demonstrated that miRNAs are involved in virtually every cardiovascular disorder, including heart failure, cardiac hypertrophy, myocardial infarction, arrhythmias, atherosclerosis, AF and peripheral artery disease.^4^, ^41^, ^42^ For example, miR‐133 and miR‐590 are both down‐regulated in a canine model of nicotine‐induced AF, leading to increased expression of TGF‐β1 and TGFβRII.^43^ Expression of miR‐29 was decreased in the left atrium of dogs with heart failure, which develop an AF‐maintaining fibrotic substrate.^7^, ^44^ In contrast, miR‐21 has been found to promote atrial fibrosis and fibrillation onset and can nearly abolish the duration of AF.^45^, ^46^ Meanwhile, miRNAs are considered important regulators of gene expression, suppressing the expression of target genes through translational repression or degradation of a target transcript^47^; miR‐328, miR‐101, miR‐433, miR‐145, miR‐122 and miR‐26a can regulate the ECM expression level by targeting TGFβRIII, TGFβRI, AZINI, JNK1, TGF‐β, TGFβRII and other matrix genes to regulate the progression of cardiac fibrosis.^26^, ^48^, ^49^, ^50^, ^51^, ^52^ We have previously shown that miR‐30, as an anti‐fibrotic microRNA, plays an important role in the control of structural changes in chronic AF.^53^ MiR‐30c has also been shown to negatively regulate cancer metastasis by directly targeting metastasis‐associated genes.^54^, ^55^ Several other studies have indicated that miR‐30c is significantly reduced in a model of pathological left ventricular hypertrophy.^7^, ^8^ MiR‐30c regulates the production of ECM in TGF‐β‐dependent liver fibrosis.^9^, ^56^ MiR‐30c reduces renal fibrosis and improves renal function in diabetic nephropathy by targeting connective tissue growth factor (CTGF).^10^ Additionally, we observed that atrial fibrosis was increased and the level of miR‐30c was decreased in left atrium following AAC, which suggesting that the alteration of miR‐30c expression may closely relate to fibrosis.

TGF‐β1 is involved in cell proliferation, apoptosis, differentiation and migration as well as the production of ECM molecules, leading to cardiac fibrosis.^15^, ^57^, ^58^, ^59^, ^60^, ^61^ TGF‐β1 is considered the major growth factor directly promoting myofibroblast development by inducing expression of α‐SMA. The transformation of fibroblasts into myofibroblasts is a critical event in the genesis of cardiac fibrosis.^62^, ^63^ During cardiac fibrosis, CFs underwent phenotypic transition to myofibroblasts marked by increased α‐SMA, which is a reliable and classic marker for the myofibroblast.^33^, ^34^, ^35^ In addition to the enhancement of contractility, myofibroblasts characteristically demonstrate increased migratory activity.^64^ CF proliferation, migration and differentiation are important factors in cardiac fibrosis. MiR‐30c has been reported to be involved in many cellular functions that lead to fibrosis, including cell proliferation, migration and differentiation.^54^, ^65^ Our study indicated that miR‐30c was regulated by TGF‐β1. Therefore, in our study, we primarily focused on the effects of miR‐30c on CF proliferation, migration and differentiation to determine the potential mechanism involved in atrial fibrosis.

Consistent with these reports, the present study confirmed that miR‐30c inhibits the transformation of CFs into myofibroblasts induced by TGF‐β1 by decreasing the expression of α‐SMA and vimentin, whereas miR‐30c inhibition promoted the expressions of α‐SMA and vimentin. In addition, miR‐30c mimic inhibited the differentiation of CFs as evidenced by a decrease in α‐SMA staining (Figure S1a). MiR‐30c inhibitor promoted CF differentiation by increasing in α‐SMA staining (Figure S1b). To the best of our knowledge, this is the first report that miR‐30c exerts its cardio‐protective properties by blocking fibroblast differentiation to myofibroblast. Our data also revealed that miR‐30c overexpression decreased cell proliferation and migration. Collectively, miR‐30c reduced CF migration, preventing the accumulation of myofibroblasts and protecting against the fibrotic process. Aberrant fibroblast proliferation and their transformation to myofibroblasts, as well as migration, are hallmarks of cardiac fibrosis, which is characterized by excessive ECM deposition and leads to distorted organ architecture and function.^66^, ^67^ Notably, miR‐30c up‐regulation was inversely correlated with the decreased expression levels of Col I and Col3α1 in cells treated with TGF‑β1. ECM metabolic imbalance is involved in various cardiovascular diseases such as myocardial infarction, heart failure and AF.^68^, ^69^, ^70^ Moreover, miR‐30c overexpression decreased the ECM‐regulated gene expression levels of Col I and Col3α1 in CFs, whereas MiR‐30c inhibition promoted these processes.

TGFβRII is a transmembrane receptor necessary for TGF‐β1 signal transduction activation. TGF‐β1 is involved in the processes of pulmonary fibrosis and liver fibrosis.^24^, ^52^ A previous study demonstrated that constitutive expression of dominant‐negative TGFβRII in the posterior left atrium resulted in a significant decrease in atrial fibrosis via inhibition of the TGF signalling pathway and attenuated fibrosis‐induced changes in atrial conduction and restitution to decrease AF.^27^ The increased level of TGFβRII in the TGF‐β1 group showed that TGFβRII may participate in TGF‐β1‐induced fibrosis. Luciferase reporter assays confirmed that miR‐30c led to a reduction in luciferase activity in TGFβRII 3′UTR‐WT group, but had no effect when miR‐30c binding site in TGFβRII 3′UTR was mutated, implying TGFβRII is a target gene of miR‐30c. The protein levels of TGFβRII were negatively correlated with miR‐30c expression in AAC model and in the cellular model of fibrosis induced by TGF‐β1. Overexpression of miR‐30c decreased the level of TGFβRII, whereas miR‐30c inhibition increased the level of TGFβRII. Therefore, TGFβRII, which is necessary for TGF‐β1 signal transduction, may represent the point at which miR‑30c inhibits the pro‑fibrotic effects of TGF‐β1.

The protective effects of miR‐30c overexpression against atrial fibrosis were confirmed by overexpressing miR‐30c via AAV9 in vivo. In our vivo study, miR‐30c overexpression markedly reduced atrial fibrosis compared with that in AAC group. We have determined for the first time that miR‐30c overexpression might significantly reduce atrial fibrosis by targeting TGFβRII expression. Although several evidences here strongly support the functional role of miR‐30c in regulating atrial fibrosis, more rigorous approaches are required to support this contention. This may include intra‐myocardial rather than systemic delivery with a CF‐specific promoter or using a miR‐30c transgenic mouse model created using the CF‐specific promoter.

**Figure 6:**
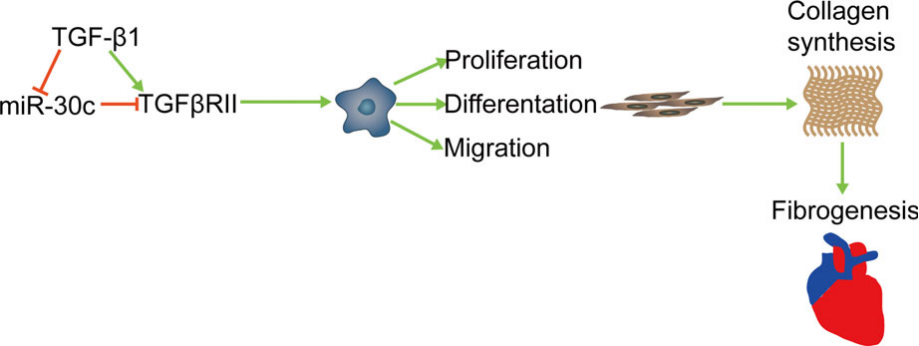
MiR‐30c regulates atrial fibrogenesis. TGF‐β1 decreased miR‐30c expression. Overexpression of miR‐30c in cardiac fibroblasts inhibited TGFβRII expression, thus preventing cell proliferation, differentiation and migration as well as collagen synthesis and atrial fibrogenesis

Collectively, miR‐30c may function by repressing TGFβRII expression, resulting in decreasing CF proliferation, differentiation, collagen synthesis and migration (Figure 6).

Unfortunately, this study was performed in a model of atrial fibrosis, which does not induce AF by burst pacing (Figure S2c). Previous study reported that P wave duration (PWD) was useful in identifying patients at a risk for paroxysmal AF and was significantly correlated with the atrial electromechanical delay.^71^ PR interval is an amalgamated measure of atrial and atrioventricular nodal conduction. PR interval prolongation increases AF risk and all‐cause mortality.^72^, ^73^ In our study, we have analysed the PWD and PR interval among the 4 groups. The results indicated that PWD and PR interval were prolonged in AAC model, while shortened in AAC + miR‐30c group (Figure S2a and b). Based on the results, these indicate that miR‐30c may decrease the risk of the occurrence of AF indirectly.

In conclusion, we suggest that TGF‐β1 produces both pro‐fibrotic and anti‐fibrotic signals and exogenous miR‐30c administration decreases atrial fibrosis by counteracting the pro‐fibrotic stimuli elicited by TGF‐β1. Our data demonstrate that overexpression of miR‐30c decreases left atrial fibrosis by inhibiting CF proliferation, differentiation, migration and collagen production by targeting TGFβRII for the first time. Our study indicates that the application of exogenous miR‐30c may be an attractive treatment strategy for atrial fibrosis and requires a further investigation in the future.

## CONFLICT OF INTERESTS

The authors declare no conflict interests.

## AUTHOR CONTRIBUTIONS

Juan Xu, Yong Wei and Haiqing Wu conceived and designed the study. Juan Xu, Songwen Chen performed the cell line experiments. Juan Xu, Haiqing Wu, Yong Wei and Baozhen Qi performed the experiments in rat models. Genqing Zhou, Liqun Zhao and Lidong Cai provided expert advice. All authors analysed the results. Juan Xu wrote the manuscript and Shaowen Liu reviewed the manuscript. All authors read and approved the final manuscript.
