# Supplementary material for: MicroRNA‐30c suppresses the pro‐fibrogenic effects of cardiac fibroblasts induced by TGF‐β1 and prevents atrial fibrosis by targeting TGFβRII
Source: J Cell Mol Med. 2018 Mar 13;22(6):3045–57. doi: 10.1111/jcmm.13548 (PMC5980214; doi:10.1111/jcmm.13548)
Supplement: Supplementary file 1 [file JCMM-22-3045-s001.doc]

**Supplementary Material**

**MicroRNA-30c suppresses the pro-fibrogenic effects of cardiac fibroblasts induced by TGF-β1 and prevents atrial fibrosis by Targeting TGFβRII**

Juan Xu^a,#^, Haiqing Wu^a,#^, Songwen Chen^a,#^, Baozhen Qi^b^, Genqing Zhou^a^, Lidong, Cai^a^, Liqun Zhao^a^, Yong Wei^c,*^, Shaowen Liu^a,*^

^a^ Department of Cardiology, Shanghai General Hospital, Shanghai Jiao Tong University School of Medicine, Shanghai, China;

^b^ Department of Cardiology, Shanghai Institute of Cardiovascular Disease, Zhongshan Hospital, Fudan University, Shanghai, China;

^c^ Department of Cardiology, Shanghai Songjiang Central Hospital, Shanghai, China;

^#^ These authors contributed equally to this work.

^*^ Correspondence: [weiyong202@sohu.com](mailto:weiyong202@sohu.com)(Y.W.); [shaowen.liu@hotmail.com(S.L.)](mailto:shaowen.liu@hotmail.com(S.L.))

**Materials and Methods**

**Immunofluorescence staining**

CFs cultured on glass coverslips were fixed in 4% paraformaldehyde for 30 minutes at 37℃. CFs were then permeabilized with 0.3% Triton X-100 in PBS for 15 minutes and blocked with 1% bovine serum albumin at room temperature for 30 minutes. Afterwards, the cells were incubated with primary antibody, rabbit anti-α-SMA (1:200, Abcam) overnight at 4℃. The slides were then rinsed and incubated with a goat anti-rabbit secondary antibody (1:200, Abcam) for 1 hour at room temperature. The slides were counterstained with DAPI (Beyotime Biotechnology, China) and then subjected to fluorescence microscopy (Leica, Germany).


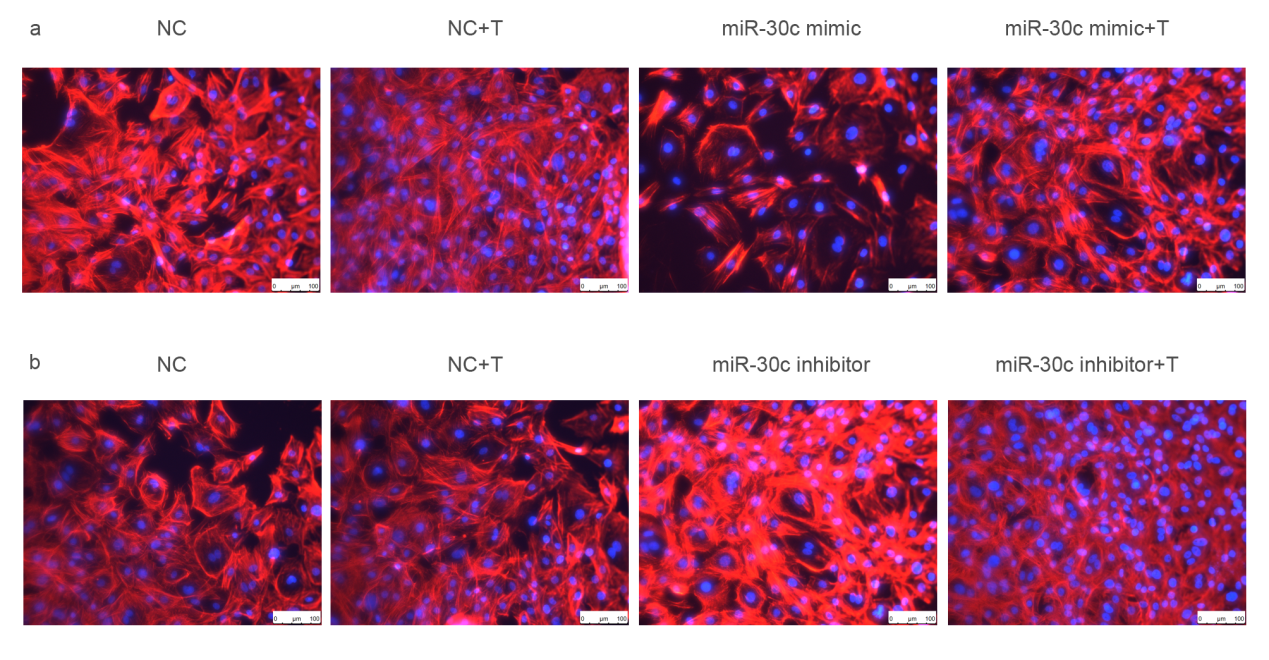


**Supplement Figure. 1 MiR-30c inhibits CF differentiation into myofibroblasts.** a. miR-30c overexpression decreased the staining of α-SMA in the absence or presence of TGF-β1 stimulation. b. miR-30c inhibitor increased the staining of α-SMA in the absence or presence of TGF-β1 stimulation (Scale bar, 100μm).

**Electrophysiological recordings**

Electrophysiological parameters were recorded and evaluated using BL-420S (TechMan, China) after AAC for 8 weeks. The rats were anesthetized with 40 mg/kg sodium pentobarbital before being intubated and placed on mechanical ventilation with a volume-cycled ALC-V8S ventilator (Alcott Biotech, China) at frequency of 70 per min with a tidal volume of 8 ml. Then, a thoracotomy was performed. The chest and pericardium was opened and the heart was clearly exposed. The ends of two electrodes were directed to the left atrial appendages. After ten minutes of stabilization, a series of electrophysiological tests were initiated. Rapid bursting stimulation at 20Hz was used to induce AF, which was defined as an irregular atrial rate lasting longer than one second. The AF induction was repeated 3 times by burst pacing. The P-wave duration (PWD) was calculated using the average of 10 P-P intervals. The PR interval was defined as the interval from the onset of the P-wave to the end of the PR segment and was calculated using the average of 10 PR intervals.


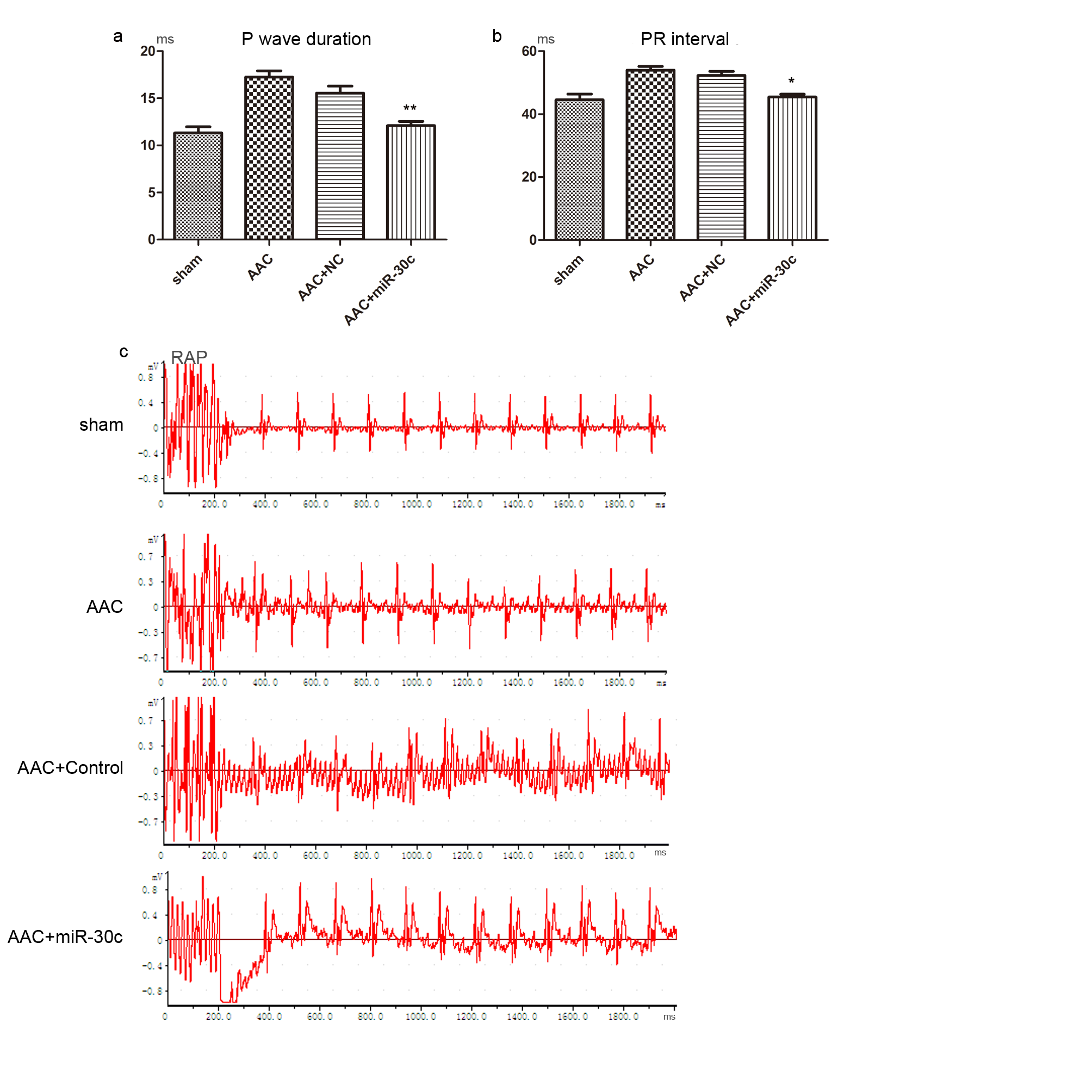


**Supplement Figure. 2. Surface electrocardiogram lead (II) analysis.** a. The P-wave duration (PWD) was calculated using the average of 10 P-P intervals. PWD was prolonged in the AAC group but shortened in the AAC+miR-30c group. ^**^P<0.01, compared with AAC group. b. The PR interval was defined as the interval from the onset of the P-wave to the end of the PR segment and was calculated using the average of 10 PR intervals. The PR interval was prolonged in the AAC group but shortened in the AAC+miR-30c group. ^*^P< .05, compared with AAC group. c. Rapid pacing of the left atrial appendage at 20 Hz (5 V, 0.2 ms) for 10 s. AF was not appear after rapid pacing in four groups. (sham, n=6; AAC, n=6; AAC+miR-30c, n=7; AAC+Control, n=7; NC=Control). The results indicate the mean ± SEM.


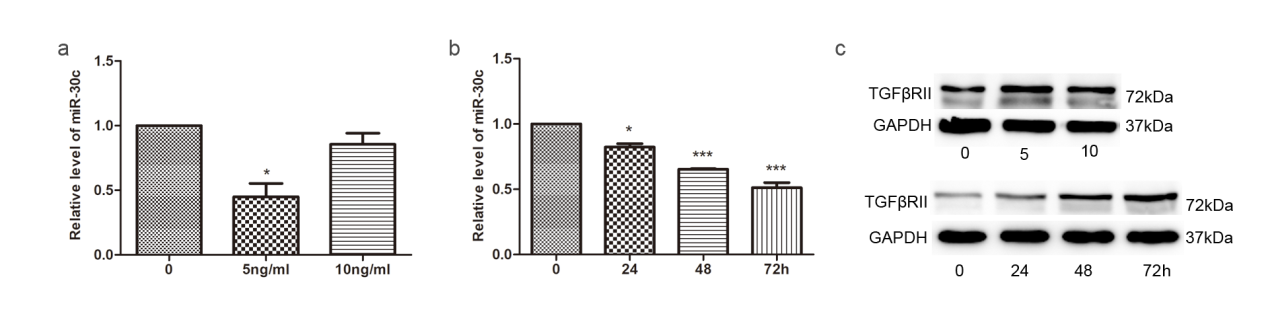


**Supplement Figure. 3 The levels of miR-30c and** **TGFβRII in TGF-β1-induced CFs.** a. The expression of miR-30c was decreased after CFs stimulated with TGF-β1 (5ng/ml) for 24 hours compared with that in control group. ^*^P< .05 compared with Control group. b. The expression of miR-30c was decreased in 24, 48 and 72 hours with time-independent effect. ^*^P< .05, ^***^P< .001 compared with Control group. c. The expression of TGFβRII was increased after stimulation with TGF-β1 in CFs.
